# A Retrospective Study of the Epidemiologic and Clinical Characteristics of COVID-19 Among Hospitalized Patients in Quanzhou, China

**DOI:** 10.1097/IM9.0000000000000048

**Published:** 2021-01-15

**Authors:** Wenhuang Chen, Yijian Lin, Hongbo Huang, Maosheng Cai, Dongheng Lin, Milong Su, Zhijun Su, Xibin Zhuang, Xueping Yu

**Affiliations:** 1 Department of Infectious Disease, The First Hospital of Quanzhou Affiliated to Fujian Medical University, Quanzhou, Fujian, China; 2 Department of Respiratory Disease, The First Hospital of Quanzhou Affiliated to Fujian Medical University, Quanzhou, Fujian, China; 3 Department of Respiratory Disease, Shishi City General Hospital, Quanzhou, Fujian, China; 4 Department of Respiratory Disease, Anxi County Hospital, Quanzhou, Fujian, China; 5 Department of Clinical Laboratory, The First Hospital of Quanzhou Affiliated to Fujian Medical University, Quanzhou, Fujian, China.; # These authors contributed equally to this work.

**Keywords:** COVID-19, SARS-CoV-2, delayed discharge, clinical characteristics, China

## Abstract

Coronavirus disease 2019 (COVID-19) has spread throughout China. However, information about COVID-19 in cities and regions outside Wuhan is limited and the indicators that predict the length of hospital stay for patients with COVID-19 are unclear. Therefore, we collected clinical data from 47 patients with COVID-19 in Quanzhou City. The median age was 38 years [interquartile range (IQR): 31–50 years], and 24 (51%) were male. There were 8 mild, 36 moderate, and 3 severe/critical cases. The median interval from exposure to disease onset was 13 days (IQR: 8–18 days). The incidence of severe/critical cases was 33% (3/10) in patients with hypertension. Common symptoms included fever (83%), cough (77%), fatigue (40%), a sore, dry throat (28%), and diarrhea (21%). One patient (2%) developed respiratory distress syndrome on day 13 of inpatient treatment. Six patients had leukopenia, 17 had elevated C-reactive protein (CRP), and 8 had lymphocytopenia and elevated lactate dehydrogenase (LDH). The median length of hospitalization was 22 days (IQR: 16-30 days). Dynamic monitoring of LDH, CRP, and neutrophil-lymphocyte ratio predicted whether length of hospitalization would exceed 21 days. Most patients presented with mild and moderate disease. Patients with hypertension were more likely to become severe or critical. Dynamic monitoring of LDH, CRP, and neutrophil-lymphocyte ratio levels can help predict delayed discharge from the hospital.

## Introduction

Coronavirus disease 2019 (COVID-19) spread throughout China, and subsequently spread globally, becoming a major pandemic.^1,2^ Until now, there has been some success in controlling the COVID-19 epidemic in China; however, the number of confirmed cases and deaths in other countries and regions continue to increase. The most common symptoms of COVID-19 are fever, cough, and fatigue, although some individuals experience only nasal congestion, rhinitis, sore throat, diarrhea, nausea, vomiting, and other atypical symptoms.^3^ Increasingly, symptoms previously considered infrequent are being reported. These include gastrointestinal manifestations, anosmia/ageusia, headaches, and myalgia.^4–6^ Children can carry high levels of the virus in the early stages of severe acute respiratory syndrome coronavirus 2 (SARS-CoV-2) infection but show relatively mild symptoms or are asymptomatic.^7^ Some patients may develop asymptomatic infection following exposure but test positive for SARS-CoV-2 nucleic acids.^8–10^ An increasing number of epidemiological investigations indicate that there is a possibility of transmission before symptom onset and by asymptomatic infected persons.^11–14^ Therefore, it is particularly important to strengthen the detection and early identification of asymptomatic infected persons.

In addition, SARS-CoV-2 nucleic acids can be detected in the feces of some patients with abdominal symptoms.^15^ Therefore, there is a possibility that SARS-COV-2 may be transmitted through contaminated water sources and via the fecal-oral route.^15,16^ The spread of SARS-CoV-2 proposed for COVID-19 and the urgent need for effective treatment strategies,^17^ including corticosteroids, antiviral nucleotide analogs, systemic interferons, monoclonal antibodies, interleukins, other immune modulators, and antibodies against SARS-CoV-2 constituents, are being researched. Additionally, there are currently more than 15 vaccine trials in progress. Currently, vaccines are being given priority, although there is still no specific therapy against COVID-19.^18^ According to the International Severe Acute Respiratory and Emerging Infection Consortium COVID-19 data, 18% of inpatients are admitted to the intensive care unit or high-care ward.^19^ Serious COVID-19 infection, in addition to the respiratory sequelae, also with cardiovascular complications (such as myocardial injury, arrhythmia, cardiomyopathy and heart failure, and acute kidney injury) and neurological complications (such as encephalopathy related to acute ischemic stroke), usually require renal replacement therapy.^20,21^ Therefore, it is important to determine the relationship between the clinical characteristics of mild and moderate COVID-19 and clinical progress.

**Figure 1:**
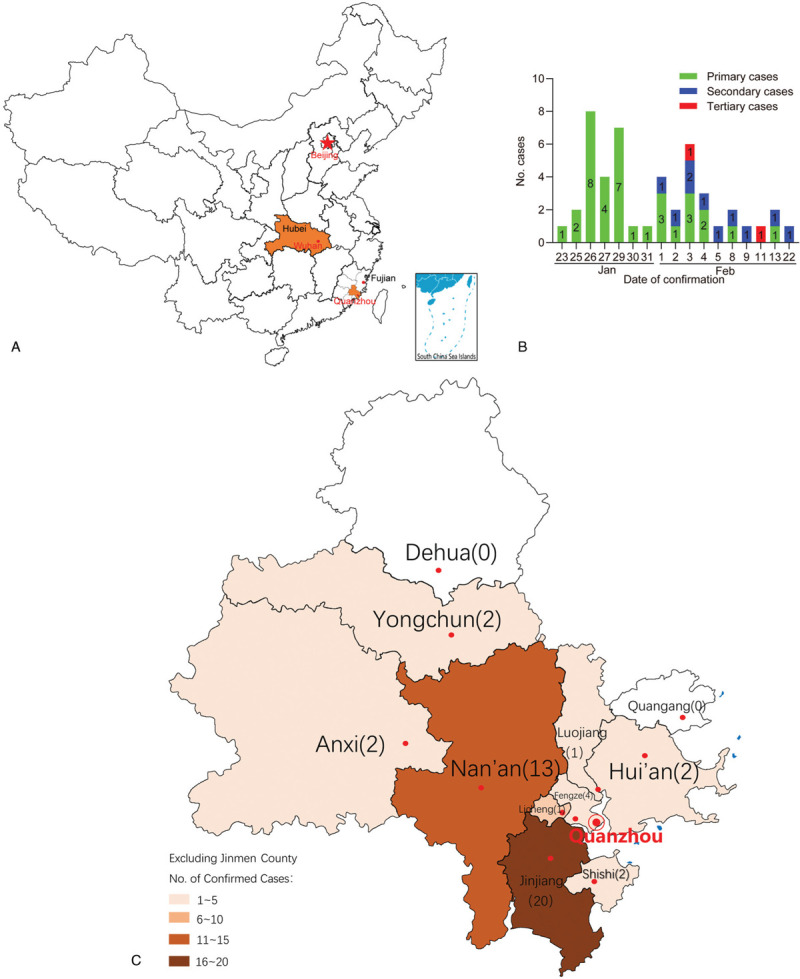
**Distribution and time series of patients with coronavirus disease 2019 (COVID-19) across Quanzhou City**. A: Location of Quanzhou and Wuhan, China. B: Time series of COVID-19 cases identified in Quanzhou City. C: Geographic distribution of the patients with COVID-19 throughout Quanzhou City, Fujian Province, China. Circles indicate capital cities.

Quanzhou, a city in Fujian Province, is located on the southeast coast of China, remote from Wuhan (Figure 1A). There are many people from Quanzhou who are engaged in trade in Wuhan or previously lived in Wuhan for a long period of time. As of January 23, 2020, the date on which the Wuhan lockdown began, the Quanzhou Center for Disease Control and Prevention (Quanzhou, China) had identified only one case of COVID-19, which was followed by an additional 21 cases being reported within the next 6 days. Within the following 3 weeks, 47 cases of COVID-19 were confirmed in Quanzhou City. Most cases were imported from Wuhan, and the majority of the patients developed mild or moderate disease. However, information about COVID-19 in cities or regions outside Wuhan is rare and the indicators that predict the length of hospital stay for COVID-19 patients is still not clear. Therefore, we collected the clinical data from patients with COVID-19 in Quanzhou to describe their clinical characteristics and dynamic profile of laboratory findings over the course of their hospitalization, to determine predictors of longer hospitalization and to provide a reference for diagnosis and treatment of COVID-19 in China and elsewhere.

## Results

### Epidemiological characteristics

We collected clinical data from 47 patients with confirmed COVID-19 admitted to the hospital between January 24 and March 6, 2020. Of these patients, 8, 36, 2, and 1 had mild, moderate, severe, and critical disease, respectively. Except for two patients with severe disease who were from Shishi City General Hospital and Anxi County Hospital, the other 45 patients were from the First Hospital of Quanzhou. As the number of severe and critical patients was low, they were combined into one group for analyses. The median age of the patients was 38 years, and 43 (91%) were aged 21–60 years. The ages of the three severe/critical patients were 35, 59, and 77 years, and all were male. There were similar numbers of male (n = 24, 51%) and female (n = 23, 49%) patients, and their ages were comparable. One (2%) patient had visited the Huanan Wholesale Seafood Market, which was associated with the first case reports, and all patients had direct or indirect contact with others confirmed to have SARS-CoV-2 infection. Most patients lived in Wuhan but had returned to Quanzhou, and 34 (72%) had spent at least 2 weeks in Wuhan; 1 patient had stopped at Wuhan for 8 h to catch a train. The remaining 12 patients (26%) had a history of direct or indirect contact with individuals confirmed to have COVID-19 before their diagnosis (Figure 1B). There were nine family clusters (n = 25, 53%) and 1 local social activity cluster (n = 9, 19%). Jinjiang and Nan’an were the areas with the most cases (n = 20, 43%, and n = 13, 28%, respectively). Figure 1C shows the geographic distribution of the patients.

### Clinical presentation

The clinical presentation of the 47 patients is shown in Table 1. Among the 15 patients who were able to provide an accurate exposure history, the median incubation period was 13 days and the longest incubation period was 24 days. Excluding one patient who had a nosocomial infection, the median interval from disease onset to hospitalization was 3 days [interquartile range (IQR): 1–6 days]. One patient was asymptomatic on admission. No patients had dyspnea on admission, although one patient developed respiratory distress syndrome on day 13 after admission. During their hospitalization, patients with severe/critical disease had a lower median partial pressure of oxygen than patients with mild and moderate disease. The median oxygenation index of severe/critical patients was also lower than that of patients with mild and moderate disease.

**Table 1:** Demographic and epidemiological characteristics of patients with COVID-19

Characteristics	All patients(n = 47)	Mild(n = 8)	Moderate(n = 36)	Severe/Critical(n = 3)
Age, Median (IQR), years	38 (31–50)	30 (23–35)	40 (31–50)	59 (47–68)
Age, years				
<20 years	2 (4.26)	2 (25.00)	0	0
21–40 years	24 (51.06)	5 (62.50)	18 (51.43)	1 (33.33)
41–60 years	19 (40.43)	1 (12.50)	17 (47.22)	1 (33.33)
>61 years	2 (4.26)	0	1 (2.78)	1 (33.33)
Sex				
Male	24 (51.06)	5 (62.50)	16 (44.44)	3 (100)
Female	23 (48.94)	3 (37.50)	20 (55.56)	0
Chronic medical illness				
Any	25 (53.19)	5 (62.50)	17 (47.22)	3 (100)
Hypertension	10 (21.28)	1 (12.50)	6 (16.67)	3 (100)
Diabetes	5 (10.64)	1 (12.50)	2 (5.56)	2 (66.67)
Respiratory disease	4 (8.51)	0	3 (8.33)	1 (33.33)
Cerebrovascular disease	1 (2.13)	0	0	1 (33.33)
Cardiovascular disease	0	0	0	0
Chronic kidney disease	2 (4.26)	1 (12.50)	0	1 (33.33)
Chronic liver disease	11 (23.40)	4 (50.00)	6 (16.67)	1 (33.33)
Malignant tumor	1 (2.13)	0	1 (2.78)	0
Previous surgery	9 (19.15)	1 (12.50)	6 (16.67)	2 (66.67)
Live in Wuhan ≥2 weeks				
Yes	34 (72.34)	5 (62.50)	27 (75.00)	2 (66.67)
No	13 (27.66)	3 (37.50)	9 (25.00)	1 (33.33)
Familial cluster	25 (53.19)	6 (75.00)	18 (50.00)	1 (33.33)
Signs and symptoms				
Fever	39 (82.98)	3 (37.50)	32 (91.43)	2 (100)
The highest temperature (°C)				
<37.3	8 (17.02)	5 (62.50)	3 (8.33)	0
37.3–38.0	22 (48.89)	3 (37.50)	18 (50.00)	1 (33.33)
38.1–39.0	16 (34.04)	0	14 (38.89)	2 (66.67)
>39.0	1 (2.22)	0	1 (2.78)	0
Breathing rate >24 times/min	0	0	0	0
Partial oxygen pressure (Quartile range, mmHg)	90.7 (79.3–104)(n = 36)	88.8 (88.35–101.5)(n = 7)	93.7 (80.8–109.1)(n = 26)	64.8 (64.3–72.4)
Oxygenation index (Quartile range)	431.5 (366.3–494.8) (n = 36)	423 (420.5–483.5) (n = 7)	441 (374.5–516) (n = 26)	304 (250–342.5)
Mean arterial pressure (Quartile range, mm Hg)	97 (87–104)(n = 46)	101 (96–104)(n = 7)	93 (86–102)(n = 36)	105 (104–111)(n = 3)
Cough	36 (76.60)	6 (75.00)	28 (77.78)	2 (66.67)
Expectoration	26 (55.32)	4 (50.00)	19 (52.78)	3 (100)
Fatigue	19 (40.43)	2 (25.00)	15 (41.67)	2 (66.67)
Aching pain	7 (14.89)	0	7 (19.44)	0
Hemoptysis	4 (8.51)	0	3 (8.33)	1 (33.33)
Headache	8 (17.02)	2 (25.00)	5 (13.89)	1 (33.33)
Diarrhea	10 (21.28)	1 (12.50)	7 (19.44)	2 (66.67)
Nausea and vomiting	2 (4.26)	1 (12.50)	0	1 (33.33)
Anorexia	6 (12.77)	0	6 (16.67)	0
Shortness of breath	9 (19.15)	0	7 (19.44)	2 (66.67)
Chest tightness/pain	11 (23.40)	0	10 (27.78)	1 (33.33)
Pharyngalgia	13 (27.66)	4 (50.00)	9 (25.00)	0
Stuffy and runny nose	7 (14.89)	5 (62.50)	2 (5.56)	0
Incubation period (Quartile range, days)	13 (8–18)(n = 15)	9 (7–14)(n = 3)	13 (8–16)(n = 11)	22(n = 1)
Time from onset to admission (Quartile range, day)	3 (1–6)(n = 46)	4 (2–9)(n = 8)	2 (1–6)(n = 36)	2 (2–3)(n = 2)
Time to admission after reentry or exposure (Quartile range, day)	10 (7–14)(n = 45)	10 (8–16)(n = 8)	10 (7–14)(n = 35)	7 (4–10)(n = 2)

The percentage is not 100% due to lack of data. Except for special instructions, the data are expressed in the number of cases (percentage, %).

### Laboratory and imaging tests

The results of the laboratory and imaging tests are shown in Table 2. On computed tomography (CT) scans, 31 patients (66%) had bilateral lung involvement; 39 (83%) had patchy or plaque-like shadows; and 29 (62%) had ground-glass opacities. In addition, 22 (47%) had air bronchograms; 12 (26%) had consolidation; 5 (11%) had a halo/reversed halo sign; and one (2%) had bat-wing opacities. The lesions of most patients (n = 30, 64%) were in the extrapulmonary region and below the pleura.

**Table 2:** Laboratory finding of patients with COVID-19

Laboratory Findings	All patients(n = 47)	Mild(n = 8)	Moderate (n = 36)	Severe/Critical(n = 3)	Normal Range
Leucocytes (∗10^9/L)	5.47 (4.40–6.48)	7.47 (5.76–8.75)	5.34 (4.33–6.2)	3.91 (2.93–4.59)	3.5–9.5
Neutrophil (∗10^9/L)	3.23 (2.43–3.87)	3.73 (3.36–4.99)	3.18 (2.33–3.81)	2.59 (1.98–3.1)	1.8–6.3
Neutrophil percentage (%)	61.1 (53.15–69.95)	56.45 (52.73–59.18)	62.45 (53.05–70.5)	68.3 (67.25–69.5)	40–75
Lymphocytes (∗10^9/L)	1.55 (1.10–1.94)	2.11 (1.83–2.95)	1.45 (1.18–1.80)	0.93 (0.71–1.02)	1.1–3.2
Lymphocytes percentage (%)	29.9 (23.50–34.75)	32.15 (30.35–34.9)	27.4 (21.93–34.8)	23.8 (22.75–24.3)	20–50
Neutrophil-to-Lymphocyte ratio	2.10 (1.56–2.92)	1.79 (1.51–1.92)	2.35 (1.54–3.09)	2.85 (2.82–3.06)	
Hemoglobin (g/L)	137 (127–151)	148 (136–152)	136 (125–148)	135 (122–146)	115–150
Platelet (∗10^9/L)	228 (190–265)	273 (237–294)	219 (190–262)	109 (107–168)	125-350
C-reactive protein (mg/L)	3.65 (0.51–14.4)	0.5 (0.49–0.52)	5.31 (1.40–14.1)	111.53 (85.69–116)	≤8
Procalcitonin (ng/ml) (percentage, %)					
≤0.1	39 (95.12) (n = 41)	5 (100) (n = 5)	32 (96.97) (n = 33)	2 (66.67)	
>0.1	2 (4.88) (n = 41)	0	1 (3.03) (n = 33)	1 (33.33)	
D-dimer (mg/L)	0.32 (0.26–0.47)	0.27 (0.21–0.31)	0.32 (0.26–0.42)	0.59 (0.56–0.75)	0–0.55
Prothrombin time (S)	11.5 (11–11.8)(n = 43)	11.3 (11–11.6)(n = 7)	11.5 (10.9–11.8)(n = 33)	11.7 (11.4–12.3)	9–13
>13	2 (4.65)	0	2 (6.06)	0	
≤13	41 (95.35)	7 (100)	31 (93.94)	3 (100)	
Troponin I (ng/ml)	0.002 (0.001–0.003)(n = 39)	0.002 (0.001–0.003)(n = 4)	0.002 (0.001–0.003)(n = 33)	0.009 (0.007–0.012)(n = 2)	≤0.026
Alanine transaminase (U/L)	23 (14–34)	31 (16–35)	19 (14–27)	24 (24–30)	7–40
Aspartate Aminotransferase (U/L)	24 (19–30)	19 (18–23)	25 (19–29)	31 (31–50)	13-35
Albumin (g/L)	39.2 (36.3–42.1)	42.7 (40.6–44.8)	38.0 (36.1–41.6)	34.9 (32.9–38.0)	40–55
Total bilirubin (μmol/L)	15.9 (11.3–23.9)	22.8 (18.0–24.6)	15.9 (10.8–23.6)	14 (10.5–14.7)	≤23
Urea nitrogen (mmol/L)	3.56 (2.93–4.08)	3.99 (3.59–4.03)	3.22 (2.81–3.81)	4.42 (4.30–5.36)	3.1–8.8
Creatinine (μmol/L)	63.1 (53.85–77.55)	69.65 (56.38–77.9)	62.8 (53.43–74.28)	63.1 (59.35–93.95)	41–81
Creatine kinase (U/L)	69 (44–103)	67 (52–76)	71 (43–107)	45 (42–228)	40–200
Lactic dehydrogenase (U/L)	172 (149–203)	145 (131–171)	173 (155–206)	292 (210–336)	120–250
Glucose (mmol/L)	5.11 (4.82–5.8)	5.00 (4.82–5.16)	5.25 (4.86–5.8)	5.81 (4.94–8)	3.89–6.11
Kalium (mmol/L)	3.74 (3.58–4.1)	3.95 (3.74–4.17)	3.71 (3.54–4.00)	4.1 (3.87–4.57)	3.5–5.3
Sodium (mmol/L)	138 (137–140)	140 (138–141)	138 (137–139)	137 (136–138)	137–147
Erythrocyte sedimentation rate (mm/h)	17 (11–22) (n = 10)	7 (n = 1)	17 (15–22) (n = 9)	NA	0–20
Pulmonary imaging (%)					
Bilateral involvement	31 (65.96)	1 (12.50)	28 (77.78)	3 (100)	
Flaky/patchy shadows	39 (82.98)	0	36 (100)	3 (100)	
Ground glass shadow	29 (61.70)	0	27 (75.00)	2 (66.67)	
Consolidation	12 (25.53)	0	12 (33.33)	0	
Pulmonary vessel thickening	34 (72.34)	3 (37.50)	30 (83.33)	1 (33.33)	
Broncho meteorology	22 (46.81)	0	21 (58.33)	1 (33.33)	
Halo/anti-halo sign	5 (10.64)	0	5 (13.89)	0	
Peripheral tape, subpleural	30 (63.83)	0	29 (80.56)	1 (33.33)	
The bat wings	1 (2.13)	0	0	1 (33.33)	
Pleural effusion	1 (2.13)	0	0	1 (33.33)	

The percentage is not 100% due to lack of data. Except for special instructions, the data are expressed in the number of cases (percentage, %).

### Clinical treatment

The details on treatment and adverse effects are shown in Table 3. Three patients (6%) were admitted to the intensive care unit for treatment, of whom one had moderate disease and two had severe/critical disease. All patients received antiviral treatment and antiviral drugs. The drugs included oseltamivir, interferon-α, lopinavir/ritonavir, ribavirin, umifenovir, chloroquine phosphate, hydroxychloroquine, and peramivir. All patients were treated with interferon-α. Of the patients, 46 (98%), 29 (62%), and 22 (47%) were treated with lopinavir/ritonavir, ribavirin, and umifenovir, respectively, and 10 (21%), 17 (36%), 13 (28%), and 7 (15%) patients were treated with 2, 3, 4, and 5 antiviral drugs concurrently. Antibacterial drugs were administered to 16 (34.04%) patients, primarily monotherapy with quinolones (moxifloxacin and levofloxacin). In addition, most patients received other treatments, including glucocorticoids (n = 13, 28%), thymalfasin (n = 19, 40%), acetylcysteine tablets (n = 39, 83%), and probiotics (n = 42, 89%). Twenty patients (43%) received oxygen therapy, one patient with moderate disease received noninvasive ventilator-assisted breathing, and two severe/critical patients (4%) received high-flow oxygen. Among the three severe/critical patients, two received high-flow oxygen therapy and one received invasive assisted ventilation and prone ventilation.

**Table 3:**
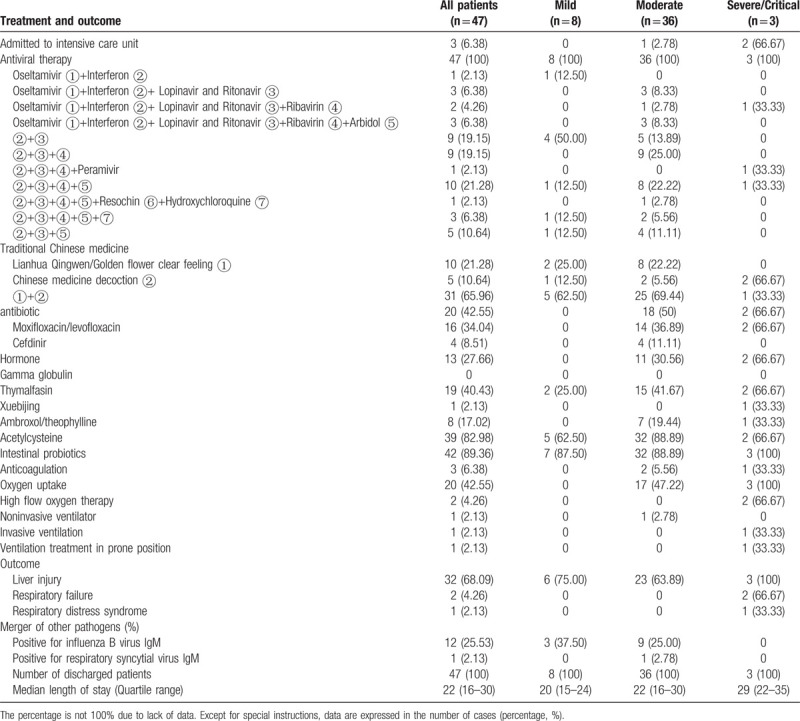
Treatment and outcome of patients with COVID-19

Adverse drug reactions included nausea (n = 7, 15%), diarrhea (n = 7, 15%), abdominal pain (n = 2, 4%), and chest tightness (n = 1, 2%). All the patients who experienced adverse drug reactions completed treatment after symptomatic treatment or drug switching. Thirty-two patients (68%) developed liver impairment, although none developed liver failure.

### Relationship between dynamic profile of laboratory finding and length of hospitalization

**Figure 2:**
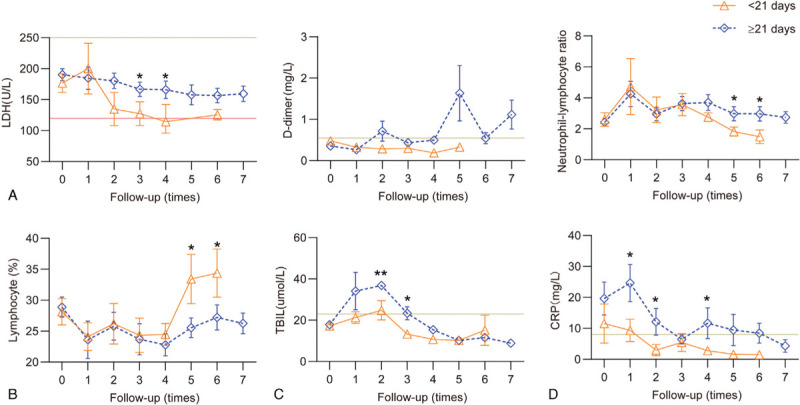
**Dynamic profile of laboratory parameters in 47 patients with coronavirus disease 2019 (COVID-19)**. Timeline charts illustrate the laboratory parameters of 47 patients with COVID-19 [27 discharge delay group (≥21 days) and 20 control group (<21 days)] every 2 ± 1 day based on the days after the onset of illness. A: Lactate dehydrogenase, D-dimer, and Neutrophil-to-Lymphocyte Ratio gradually increased in the delayed discharge group during treatment and were higher than in the control group. B: Lymphocyte percentage was maintained at baseline levels in the group with delayed discharge, and was lower than the control group. C: total bilirubin (TBil) levels in both groups gradually increased, but the increase was greater in the delayed discharge group. D: The C-reactive protein (CRP) levels of the delayed discharge group were higher than that in the control group on admission; however, the CRP levels were the same in both groups on the 3rd follow-up. The solid lines in gray show the upper and lower normal limit of each parameter, and the solid line in red shows the lower normal limit of each parameter. ^∗^*P* < 0.05 for discharge delay group versus control group.

All 47 patients recovered and were discharged. The median length of hospitalization was 22 days; however, this varied according to the severity of the disease and was longer in patients with severe/critical disease (median: 29 days) than in those with mild (median: 20 days) and moderate (median: 22 days) disease. In the analysis of factors related to prolonged hospitalization, there were no significant differences in lactate dehydrogenase (LDH), D-dimer, neutrophil-to-lymphocyte ratio (NLR), lymphocyte percentage, C-reactive protein (CRP), or total bilirubin (TBil) between the two groups on admission. However, LDH, D-dimer, and NLR gradually increased during treatment in the delayed discharge group (≥21 days) and were higher than in the control group (<21 days) (Figure 2A), while lymphocyte percentage was maintained at baseline levels in the group with delayed discharge and was lower than the control group (Figure 2B). TBil levels in both groups gradually increased, although the increase was greater in the delayed discharge group (Figure 2C). The CRP levels of the delayed discharge group were higher than that of the control group on admission; however, the CRP levels were the same in both groups on the third follow-up (Figure 2D).

## Discussion

This retrospective study provides a detailed description of the epidemiology, clinical characteristics, laboratory and imaging test results, and treatment of 47 patients with confirmed COVID-19 in Quanzhou and provides insights into factors associated with delayed discharge. All except three patients had mild or moderate disease, and there were no deaths. These findings are similar to the findings in most regions of China other than Wuhan.^22,23^ The proportion of critical patients was lower than observed for Wuhan during the initial stage of the epidemic.^1,24^ Most of the cases were imported from Wuhan. The geographic distribution in Quanzhou reflects the geographic distribution of people who had spent time in Wuhan for business and work. There were similar numbers of males and females affected; however, the three severe/critical patients were all male, which is consistent with previous studies.^25^ The greater severity in males may be due to the protective effects of the X chromosomes and sex hormones in females.^26^ In addition, more than half of the patients (25/47, 53%) had various underlying diseases; this was especially the case in those with severe/critical disease. For example, 30% (3/10) of patients had hypertension, which may be related to the high expression of angiotensin-converting enzyme 2, the receptor of SARS-CoV-2 in patients with hypertension.^27,28^

The lymphocytes of severe/critical patients were significantly decreased, demonstrating that SARS-CoV-2 can cause T cell exhaustion similar to that observed in patients with Ebola virus disease.^29^ This provides a theoretical basis for the use of thymalfasin and other immune-boosting drugs in the treatment of severe/critical COVID-19. Consistent with previous studies,^30,31^ the lung CT scans of most patients in this study showed patchy shadows and ground-glass opacities. Therefore, combining CT scans with clinical presentation and hematological tests is useful for diagnosis,^30,32^ especially in settings where the availability of SARS-CoV-2 nucleic acid testing is limited and when clinical outcomes need to be determined.^33^

In this study, all patients had good outcomes and there were no deaths. However, the length of hospitalization varied. Patients with a delayed discharge had a persistently low lymphocyte percentage, while LDH, D-dimer, and NLR gradually increased and were higher than in patients with shorter hospitalization. Similar results were observed in patients with COVID-19 who did not survive.^3^ In addition, the CRP levels of the delayed discharge group were persistently higher than those in the control group. These test markers, which are the most useful for the clinical management of patients with COVID-19, may predict the discharge of patients with COVID-19.

Currently, there is no specific treatment for COVID-19. In addition to supportive and symptomatic treatment, and psychological intervention, it is important to carry out quarantine measures, control infection sources, cut off possible transmission routes, and increase public awareness. In this study, all patients were administered antiviral treatment. Most patients were administered gut probiotics and traditional Chinese medicine, and some patients received antibacterial drugs, steroids, and immune-boosting drugs. In addition, 39 patients were administered acetylcysteine. The antioxidant effects of acetylcysteine are thought to reduce cytotoxicity; however, the effects of acetylcysteine require further study. As many patients developed liver impairment during the course of the disease, it is necessary to monitor liver function and to administer hepatoprotective drugs (such as reduced glutathione) or to try to avoid excessive use of hepatoxic drugs (such as ribavirin).

There are some limitations of this study. First, the sample size was small and it only included patients from Quanzhou; thus, it may not comprehensively describe the clinical characteristics of those with COVID-19. Second, most patients were permanent residents of Wuhan who had returned to Quanzhou; therefore, we were unable to calculate the incubation period accurately. Third, we did not analyze the relationship between length of hospitalization and viral load, immune function, and other markers. Fourth, all patients improved and were discharged, and we were unable to assess the prognostic risk factors. Last, we were unable to compare the clinical characteristics with those of patients with COVID-19 in other countries.

In conclusion, patients with COVID-19 in Quanzhou had a lower severe/critical ratio and higher discharge rate, which differed from those found in patients from Wuhan. We also found that patients with hypertension tended to develop more severe disease. Dynamic monitoring of LDH, D-dimer, NLR, and CRP levels can help to predict the length of hospitalization. In countries throughout the world, the suddenness and magnitude of the COVID-19 pandemic has created a demand for hospitalization that exceeds hospital capacity; therefore, it is useful to be able to predict which patients are likely to require prolonged hospitalization.

## Materials and methods

### Patients and methods

We conducted a retrospective study to analyze the clinical characteristics of confirmed cases of COVID-19 treated in 3 designated grade II A and above hospitals in Quanzhou in Fujian Province.

### Population and setting

Quanzhou (117°25’E-119°05’E, 24°30’N-25°56’N) is located on the southeast coast of Fujian Province, on the west coast of the Taiwan strait (Figure 1A).^34^ Quanzhou has a population of 8.6 million and includes four districts and eight counties (Figure 1C). When the first case of COVID-19 was imported to Quanzhou, the Quanzhou government took active measures to isolate patients with COVID-19 and to trace contacts to minimize opportunities for further transmission in the community.

### Source of data

All confirmed cases fulfilled the diagnosis and treatment guidelines for COVID-19 issued by the Chinese National Health Committee and were classified as mild, moderate, and severe/critical.^35^ The classification criteria were as follows: mild–mild clinical symptoms and no imaging presentation of pneumonia; moderate–fever and respiratory symptoms present, imaging presentation of pneumonia; severe–any of the following criteria: (1) shortness of breath, respiratory rate ≥30 breaths/min; (2) finger oxygen saturation ≤93% at rest; and/or (3) partial pressure of arterial oxygen/inhaled oxygen concentration ≤300 mm Hg or lung imaging showing >50% progression of lesions within 24 to 48 h; and critical with any of the following criteria: (1) respiratory failure requiring mechanical ventilation; (2) shock; and/or (3) failure in other organs necessitating treatment in the intensive care unit.

This study was approved by the Fujian Center for Disease Control (Min Ji Kong Lun Shen 2020 No. 001) and the ethics committee of First Hospital of Quanzhou (Quan Yi Lun Shen 2020 No. 124). All patients signed the informed consent form.

### Laboratory tests

The QuantStudio 5 Real-Time PCR System (Applied Biosystems Inc., Waltham, MA, USA) was used to measure the SARS-CoV-2 nucleic acid load in sputum or pharyngeal swabs. Every patient was tested at least twice after admission, with an interval of at least 24 h between tests. An automated hematology analyzer (Coulter LH750, Beckman Coulter Inc., Brea, CA, USA) was used to measure hemoglobin and the counts of white blood cells, red blood cells, and platelets. An automated biochemistry analyzer (Beckman LX-20, Beckman Coulter Inc.) was used to measure aspartate aminotransferase, alanine aminotransferase, albumin, and TBil using the reagents provided by the manufacturer. A coagulation analyzer (ACL TOP700, Sysmex Inc., Kobe, Japan) was used to measure prothrombin time and D-dimer. An automated immunology analyzer (ARCHITECT i1000SR, Abbott Inc., Abbott Park, IL, USA) was used to measure troponin I. An automated chemiluminescence analyzer (MINI VIDAS, bioMérieux, Marcy, l-Étoile, France) was used to measure procalcitonin.

To determine the relationship between various laboratory parameters during COVID-19 progression and clinical outcomes, we repeated the laboratory tests (including hematology, biochemistry, and coagulation function) approximately every 2 days. Patients had a mean of 7 follow-up sets of laboratory tests.

### Clinical treatment

The patients received varying combinations of antiviral treatments (usual dose: interferon-α 50 μg, nebulized, 2 ×/day; lopinavir/ritonavir 500 mg 2 ×/day; ribavirin injection 500 mg 4 ×/day, ribavirin tablets 0.15 mg 4 ×/day; umifenovir 200 mg 3 ×/day; and peramivir injection 300 mg 4 ×/day). Most patients received probiotic treatment (usually: *Clostridium butyricum* and *Enterococcus* 400 mg 3 ×/day). Some patients received a 3- to 5-day course of glucocorticoid treatment (methylprednisolone succinate injection 40 mg 4 ×/day), and one critical patient received hydrocortisone by intravenous pump, methylprednisolone succinate intravenous infusion, and methylprednisolone tablets. Some patients received antibiotics, which were primarily quinolones (usual dose: levofloxacin 0.5 g/day or moxifloxacin 0.4 g/day). All patients were discharged, and the discharge criteria conformed to the *Diagnosis and Treatment Protocol for Novel Coronavirus Pneumonia (Trial Version 6)*.^35^

### Statistical analyses

All analyses and graphs were generated by using SPSS 25.0 (IBM Corp., Armonk, NY, USA) and GraphPad Prism 8.1 (GraphPad Software Inc., San Diego, CA, USA). Continuous variables were expressed as medians and IQRs. Categorical variables were expressed as percentages. The *t*-test or nonparametric Mann-Whitney *U* test was used for intergroup comparisons. *P* values <0.05 were regarded as statistically significant. To study factors related to prolonged hospitalization, we divided the patients into two groups according to whether they were hospitalized for <21 days or for ≥21 days, as described in a previous study.^36^

## Acknowledgments

The authors thank all the medical staff in the Department of Infectious Diseases and the Department of Respiratory Disease of the First Hospital of Quanzhou Affiliated to Fujian Medical University, for collecting the clinical specimens.
